# Harnessing immunomodulation during DNA damage in Ewing sarcoma

**DOI:** 10.3389/fonc.2022.1048705

**Published:** 2022-11-22

**Authors:** Jessica D. Daley, Adam C. Olson, Kelly M. Bailey

**Affiliations:** ^1^ Department of Pediatrics, Division of Pediatric Hematology and Oncology, University of Pittsburgh School of Medicine, Pittsburgh, PA, United States; ^2^ Department of Radiation Oncology, University of Pittsburgh School of Medicine, Pittsburgh, PA, United States; ^3^ Cancer Immunology and Immunotherapy Program, UPMC Hillman Cancer Center, Pittsburgh, PA, United States

**Keywords:** Ewing sarcoma, radiation, immunobiology, DNA damage, immunomodulation, relapse

## Abstract

Ewing sarcoma is a fusion-oncoprotein-driven primary bone tumor most commonly diagnosed in adolescents. Given the continued poor outcomes for patients with metastatic and relapsed Ewing sarcoma, testing innovative therapeutic approaches is essential. Ewing sarcoma has been categorized as a ‘BRCAness’ tumor with emerging data characterizing a spectrum of DNA damage repair defects within individual Ewing tumors, including the presence of EWSR1::FLI1 itself, recurrent somatic mutations, and rare germline-based defects. It is critical to understand the cumulative impact of various DNA damage repair defects on an individual Ewing tumor’s response to therapy. Further, in addition to DNA-damage-directed therapies, subsets of Ewing tumors may be more susceptible to DNA-damage/immunotherapy combinations given the significant cross-talk between DNA damage and inflammatory pathways in the tumor microenvironment. Here we review potential approaches utilizing DNA-damaging agents as modulators of the Ewing tumor immune microenvironment, with a focus on radiation and opportunities during disease metastasis and relapse.

## Introduction

Ewing sarcoma is the second most common bone tumor diagnosed in adolescents and young adults. Ewing sarcoma is driven by a fusion oncoprotein derived from the translocation of *EWSR1* on chromosome 22 with an ETS family member, most commonly *FLI1* on chromosome 11 (1). Patients with upfront metastatic or relapsed Ewing sarcoma continue to have very poor outcomes (2), and new therapeutic approaches continue to be in high demand. The exquisite sensitivity of Ewing tumors to DNA damage has been recognized for decades and DNA damaging agents such as chemotherapy and radiation continue to be the mainstays of Ewing sarcoma therapy, even for aggressive disease (3).

DNA damage can elicit significant alterations in tumor biology, including modulation of the tumor immune microenvironment (TIME). Historically, the impact of DNA damage on the Ewing TIME has been understudied given a paucity of tumor biopsies at the time of relapse and the lack of syngeneic or transgenic (immunocompetent) mouse models of Ewing sarcoma (4). DNA-damaging agents can promote immunogenicity through multiple mechanisms including increasing the neoantigen repertoire, increasing antigen presentation, and shifting the cytokine profile to promote an inflamed tumor microenvironment (5, 6). Understanding TIME alterations elicited by DNA damage specifically in Ewing sarcoma is a high priority, as TIME modulation during DNA damage may offer a new avenue for therapy for patients with aggressive disease. Therapeutically, it can be challenging to increase chemotherapy doses or add additional marrow-suppressive agents into existing chemotherapy backbones for the treatment of Ewing sarcoma, also highlighting why multi-modality approaches, such as TIME modulation, are in need.

Immunotherapy includes medications and cell-based therapies that broadly act by enhancing the anti-tumor immune response through various mechanisms (7) and have been utilized successfully in many adult carcinomas and soft tissue sarcomas (8, 9) (10). Clinical trials investigating single-agent immunotherapy, such as PD1 inhibition, have not demonstrated a significant clinical benefit in advanced Ewing sarcoma (11, 12). Given the importance of immunotherapy type and timing in disease response (13) such results are neither surprising nor discouraging when currently so little is known about the Ewing TIME. Primary Ewing sarcoma is known to have low overall immune infiltration compared to other tumors types. However, some studies have demonstrated a correlation between increased infiltration of CD8+ T cells and improved outcomes (14, 15). Our recent work demonstrated the Ewing TIME can evolve and demonstrate enhanced immune cell infiltration upon disease metastasis and relapse, possibly due to a combination of prior chemotherapy exposure and changes in tumor microenvironments (bone versus lung) (16). This work again highlights the need to better understand Ewing tumor immunobiology, especially in the setting of relapse.

In this mini-review we will discuss the layers of DNA damage repair defects in Ewing sarcoma, how DNA damaging agents can influence the TIME, and ways in which immunomodulation during DNA damage could provide new therapeutic opportunities for Ewing sarcoma in the future.

## DNA damage and Ewing sarcoma

### EWSR1::FLI1 and DNA damage sensitivity

Ewing tumors demonstrate high sensitivity to DNA damage. DNA damaging agents, including doxorubicin and cyclophosphamide, have formed the chemotherapy backbone for the treatment of Ewing sarcoma since the first use of adjuvant therapy in the 1970s (17). Ewing sarcoma is also sensitive to radiation therapy (18). Decades later, a screen of hundreds of cancer cell lines seeking to identify biomarkers for targeted cancer agents discovered EWSR1::FLI1 was significantly associated with sensitivity to the PARP [Poly (ADP-ribose) polymerase] inhibitor (PARPi) olaparib (19). PARP1 is an enzyme involved in DNA damage repair and a drug target in BRCA-mutant cancers deficient in homologous recombination repair (20). PARP1 drives transcription and accelerates base excision repair (21, 22), and inhibition of PARP1 leads to cell death in cancers deficient in homologous repair by causing defects in the replication fork needed to repair DNA damage. Further studies elucidated that EWSR1::FLI1 interacts directly with PARP (20). Gorthi et al. demonstrated that expression of the EWSR1::FLI1 fusion oncoprotein correlated with increased chemosensitivity (23). Mechanistically, they found that EWSR1::FLI1 promotes R-loop accumulation, and ultimately deranges DNA damage repair machinery by impairing normal BRCA1 functionality. A study in 2002 by Spahn et al. also demonstrated that the N-terminal portion of EWSR1::FLI1 can interact with the C-terminal portion of BRCA1-Associated Ring Domain 1 (BARD1), thus providing another potential link between EWSR1::FLI1 and BRCA1 biology (24). Such studies provided rationale for phase II clinical trial of olaparib as single-agent therapy in patients with refractory Ewing sarcoma (25) and subsequent studies have demonstrated that sensitivity to PARP inhibition in Ewing sarcoma is increased in the setting of other DNA damaging agents (irinotecan, temozolomide) (26). Despite this, the overall clinical response of Ewing tumors to PARPi has been underwhelming. Lastly, elegant work has demonstrated the importance of the level of EWSR1::FLI1 fusion oncoprotein expression on Ewing cell behavior. EWSR1::FLI1 expression can vary between cells within a tumor. It is plausible that Ewing cells with low versus high EWS::FLI1 expression may demonstrate altered sensitivity to DNA damage (27–29), thus allowing for tumor cell subpopulation targeting.

### Somatic and germline variants in Ewing sarcoma

In addition to DNA-damage-repair defects imparted by EWSR1::FLI1 in all Ewing tumors, there is the potential for Ewing tumors to harbor additional defects in DNA damage repair through the presence of somatic and germline variants or post-transcriptional modifications resulting in loss of protein expression. When comparing Ewing tumors to adult carcinomas, and even other pediatric primary bone tumors such as osteosarcoma, Ewing sarcoma demonstrates a very low tumor mutational burden (30–32). A handful of recurrent somatic variants, such as *STAG2*, *CDKN2A*, and *TP53* have been reported in Ewing sarcoma (31, 32). Ewing tumors harboring one or more of these somatic mutations may demonstrate altered responses to DNA damage, as each of the corresponding proteins have been shown to participate in DNA damage repair through different mechanisms. For example, *in vitro* studies of STAG2-deficient glioblastomas demonstrated increased sensitivity to PARP inhibition (33). In Ewing sarcoma, loss of STAG2 expression can be secondary to *STAG2* somatic mutations or loss of protein expression in the absence of a mutation (34).

**Figure 1:**
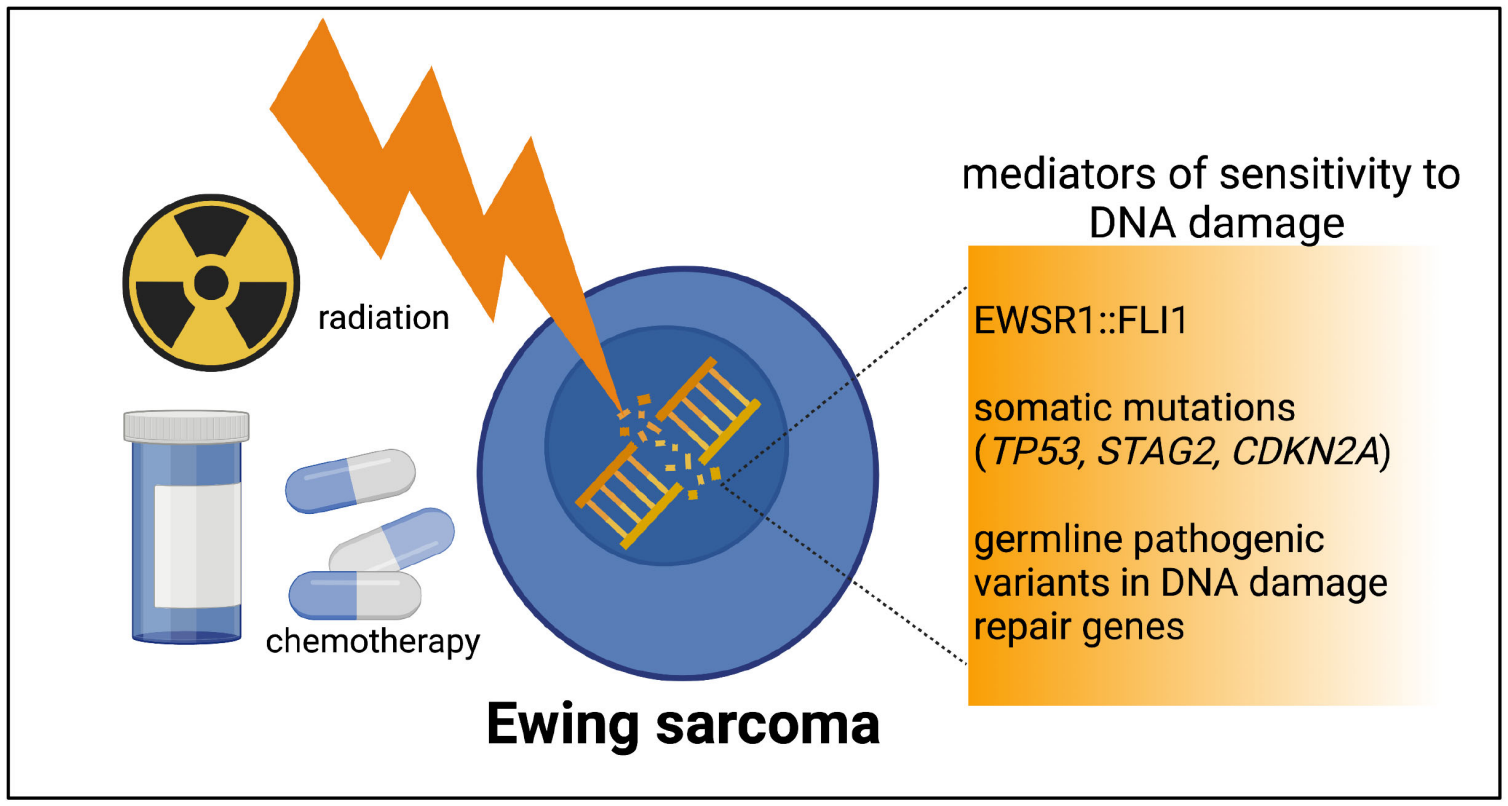
The spectrum of DNA damage repair deficiencies in Ewing tumors. Ewing tumors all have a level of DNA damage repair deficiency imparted by EWSR1::FLI1. The presence of one or more recurrent somatic mutations or rare germline pathogenic variants have the potential to contribute an additional level of DNA damage repair deficiency in a subset of Ewing tumors. Figure created by biorender.com.

A third layer of DNA-damage-repair deficiency to consider in Ewing sarcoma derives from germline pathogenic variants. Multiple sequencing studies of pediatric cancers have noted a small fraction of germline pathogenic variants in patients with Ewing sarcoma (35, 36). In a germline variant analysis of sequencing data from 175 patients with Ewing sarcoma, likely pathogenic variants were identified in 13.1% of patients (37). In the variants found, involving 22 different genes, a strong enrichment for DNA repair pathways and DNA double-strand break repair was noted on pathway analysis. Our work and others continue to add to the growing number of germline variants in DNA damage repair genes noted in patients with Ewing sarcoma (38, 39). Our group’s prior work demonstrated that loss of additional DNA damage repair machinery, such as BARD1 expression, can indeed confer Ewing cells more susceptible to DNA damage as compared wuth the sensitivity imparted by the presence of EWSR1::FLI1 alone (40). **Figure 1** depicts a brief summary of the spectrum of DNA damage repair deficiencies in Ewing sarcoma.

### DNA damaging agents used in Ewing sarcoma therapy

Given the spectrum of DNA damage repair defects in Ewing sarcoma, DNA damaging agents will continue to be a mainstay of therapy. Following the original treatment schema with doxorubicin and cyclophosphamide discussed above, in the 1980s it was noted that ifosfamide and etoposide, which also exert their anti-neoplastic effect through induction of DNA damage, were effective in treating patients with relapsed Ewing sarcoma (41). This led to the development of the National Cancer Institute protocol INT-0091 (CCG-7881 and POG-8850)

in which ifosfamide and etoposide were added to the standard therapy backbone. Improved overall and event free survival was seen in patients with newly diagnosed, localized Ewing sarcoma using this five-drug approach (42). Alternating cycles of VDC (vincristine, doxorubicin, cyclophosphamide) and IE (ifosfamide and etoposide) thus remain the standard of care for patients with upfront localized or metastatic Ewing sarcoma. AEWS0031 later demonstrated that shortening the time between cycles (interval compression) provides additional benefit (43).

In addition to chemotherapy, radiation is also an important component of Ewing sarcoma treatment. Radiation is a curative-intent treatment modality option for local control, either as definitive treatment or as adjuvant treatment following surgical resection. The commonly prescribed radiation dose for definitive treatment of primary tumors is 55-60 Gy in 1.8-2 Gy fractions (44). The most recent Children’s Oncology Group protocols for Ewing sarcoma recommend 45 Gy be delivered to the original tumor volume with an additional 10.8 Gy boost delivered to the post-induction chemotherapy volume (3). Gross residual disease post-surgical resection is treated with 55.8 Gy, and microscopic disease treated with 50.4 Gy. There has recently been data suggesting that dose escalation up to a total dose of 70.2 Gy may be of benefit in improved local control (45), although this strategy has not been widely adopted to date. More recent studies show that hypofractionation (5-10 Gy doses over 5-10 fractions) may be as or more effective at treating sarcomas, including Ewing sarcoma (46).

Radiation therapy is also a key component in the treatment of metastatic and relapsed Ewing sarcoma. For patients presenting with pulmonary metastases at diagnosis, there have been multiple studies demonstrating the survival benefit of whole lung irradiation after completion of chemotherapy (47). For patients presenting with bony metastases, outcomes are worse overall; however, radiation delivery to sites of metastatic disease is beneficial (48). Patients with solitary bone metastases benefited most from radiotherapy, with doses of up to 50 Gy to sites of bony metastases being utilized. As patients with Ewing sarcoma receiving radiation are often a higher-risk patient population (incomplete resections, metastatic disease, relapse, etc.), this is a group of high interest when considering immunotherapy interventions following post-DNA damage modulation of the Ewing TIME.

## Immunomodulation through DNA damage

### Immunomodulation by chemotherapy

DNA damaging chemotherapeutic agents have been shown to induce immunogenicity through a variety of mechanisms (5). Given the low mutational burden of Ewing sarcoma, the mutagenic potential of DNA damaging agents is an appealing mechanism of enhancing immunogenicity by production of tumor neoantigens (49). Tumor neoantigens can induce increased anti-tumor T cell response which is again beneficial when combined with immunotherapy agents. However, increased neoantigens in the TIME are not always sufficient to induce immune response (50). DNA damaging agents additionally lead to release of damage associated molecular patterns (DAMPs) after cell death. DAMPs stimulate the recruitment of antigen-presenting cells to the site of cell death and further prime the TIME for an adaptive immune response. Doxorubicin and cyclophosphamide are utilized in the treatment of Ewing sarcoma and are known to induce immunogenic cell death (51). Cyclophosphamide additionally remains of particular interest as it has been shown to increase antigen presentation on tumor cells and expand dendritic cell populations that can promote T cell priming (52, 53).

An additional mechanism by which DNA damaging chemotherapeutics can increase anti-tumor immune response is through changes in the cytokine profile of the tumor environment. Cellular response to DNA damage includes activation of signaling pathways that lead to release of proinflammatory cytokines including IFN-α and cytokines triggered by activation of the NF-κB signaling pathway. Specifically, cyclophosphamide has been shown to induce IFN-γ and IL-2, pro-inflammatory cytokines that promote immunogenicity (53). Parkes et al. demonstrated that in a breast cancer model DNA-damage-repair defects lead to increased expression of the chemokines CXCL10 and CCL5 from tumor cells (16, 54).

The precise impact of chemotherapy commonly used in relapsed Ewing sarcoma [irinotecan and temozolomide (IT), topotecan and cyclophosphamide (TC), high dose ifosfamide (IFOS), and gemcitabine and docetaxel (GD) (55)] on Ewing tumor immunobiology is still largely unknown. PARP inhibitors have been shown to induce infiltration of CD8+ T cells in breast cancer, and the efficacy of PARP inhibition is due to recruitment of these cytotoxic T cells through the cGAS/STING pathway (56). In this model, depletion of CD8+ T cells decreased the efficacy of PARP inhibition. In addition to recruiting cytotoxic T cells, PARP inhibition has also been shown to increase the expression of immune checkpoint ligand PD-L1 on cancer cells (57). Our work has previously shown that PD-L1 and PD-L2 expression can be manipulated in Ewing cell subpopulations in response to inflammatory signaling (58).

In summary, the effect of DNA damaging chemotherapeutic agents used in the treatment of Ewing sarcoma can, in theory, manipulate the TIME; however, this is an understudied area. While chemotherapy has the temporary ability to alter the TIME, ultimately due to systemic effects, patients are largely overall immunosuppressed during therapy. Thus, focal delivery of DNA damage, such as radiation therapy, may be beneficial when considering immunotherapy combinations.

### Immunomodulation by radiation

The interest in the immune-mediated effects of radiation date back to the 1980s when it was first noted that local radiation can lead to anti-tumor effect at distant sites of disease (59, 60). Subsequently, many studies have demonstrated that local radiation can produce systemic immune-mediated anti-tumor effects, though this is not a consistent finding in all studies (6, 61, 62). Studies examining the radiation anti-tumor effect in immunocompetent vs immunodeficient mouse models of melanoma have demonstrated that the presence of CD8+ cytotoxic T cells are necessary for this response (63). Radiation enhances the immune response to tumors through release of cytokines and chemokines in the tumor microenvironment following cell death (64). These cytokines and chemokines result in infiltration of effector immune cells (dendritic cells, macrophages, cytotoxic T cells) as well as immunosuppressive populations (Tregs, myeloid-derived suppressor cells) (65). Similar to the effect of chemotherapy described above, radiation induces immunogenic cell death leading to release of DAMPs. This leads to increased production and recruitment of proinflammatory cytokines and chemokines, including CXCL9, CXCL10, and CXCL11 (66). The generation of this proinflammatory environment is thought to lead to recruitment of effector T cells and may enhance the priming of T cells in the TIME. Recently, the essential role of natural killer (NK) cells in controlling the radiation-induced anti-tumor has been demonstrated (67).

In addition to promoting a proinflammatory TIME, radiation can also exert immunosuppressive effects. Tregs are a well described subset of CD4+ T cells that exert immunosuppressive effects on the TIME. In some adult carcinomas, radiation has been shown to increase Tregs and the subsequent production of immunosuppressive cytokines including TGF-β and IL-10 (68) TGF-β is known to be increased following radiation and is converted from its latent to active form by reactive species generated during radiation (69). TGF-β exerts immunosuppressive effects on the TIME and it has been shown that increase in TGF-β in the TIME can lead to decreased efficacy of immunotherapy through the exclusion of CD8+ T cells (70). Several studies have demonstrated that inhibition of the immunosuppressive pathways activated by localized radiation can improve radiation-induced tumor kill and anti-tumor immunity (71, 72).

An additional immunosuppressive cell population that can be induced/increased following radiation are myeloid-deprived suppressor cells (MDSCs). MDSCs are well described to promote tumor growth and survival and are known to be recruited into the TIME of pancreatic and prostate cancer immediately following radiation (73), with a decrease in this population seen at 1-2 weeks post radiation. TGF-β is known to induce differentiation of macrophages to an M2 phenotype which is protumor and immunosuppressive. These mechanisms of immunosuppression induced by radiation represent potential targets to improve the anti-tumor immune response induced by radiation.

## Radiation therapy in Ewing sarcoma: Untapped potential for multi-modality therapies

**Figure 2:**
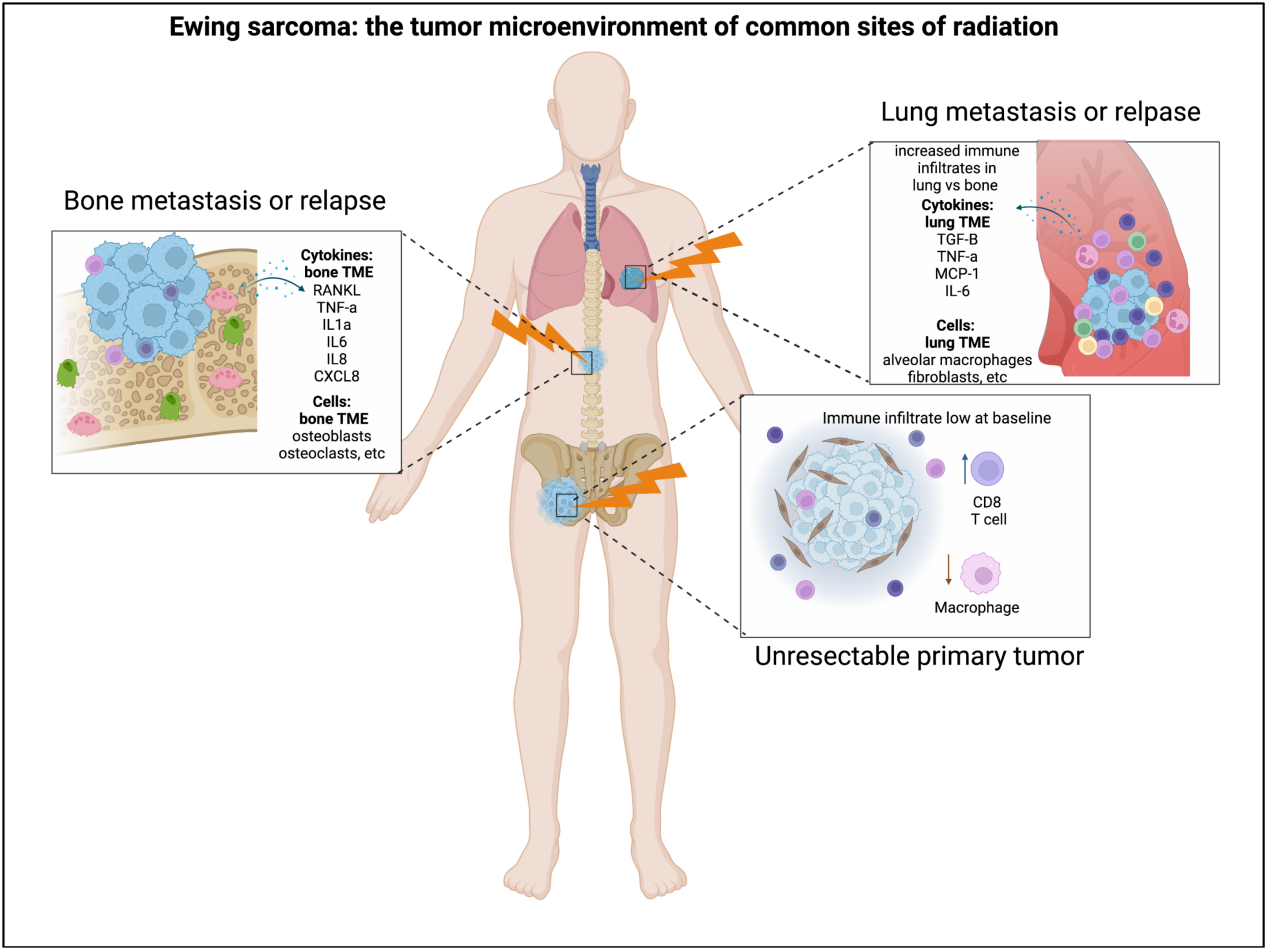
Radiation and the Ewing sarcoma tumor immune microenvironment. Radiation is often utilized for the treatment of Ewing sarcoma in the setting of unresectable primary tumors, lung metastases, relapse to the bone, etc. The Ewing tumor immune microenvironment can demonstrate differences in immune infiltration and cytokine abundance in these distinct microenvironments where radiation is utilized. Figure created by biorender.com.

Currently, relatively little is known about the specific impact of radiation on the Ewing sarcoma TIME. New therapeutic approaches for patients with metastatic and relapsed Ewing sarcoma are long overdue. While agents that induce tumor DNA damage clearly provide some benefit for the treatment of relapsed disease, they are rarely curative. Understanding which multi-modality therapeutic approaches may circumvent Ewing tumor cell resistance to single- modality therapies is a priority. It is possible that subsets of Ewing tumors in the DNA-damage- repair deficiency spectrum (**Figure 1**) could demonstrate differential responses to multi-modality therapy. Radiation therapy is often utilized in patients with high-risk (metastatic and relapsed) Ewing sarcoma, and given its potential to modulate the TIME, it is a logical treatment modality to consider in combination with immunomodulation (**Figure 2**). There has been growing interest in oncology to combine radiation with immunomodulatory agents to improve the anti-tumor immune response (74–76). Given the concurrent immune-stimulatory and immunosuppressive effects that radiation therapy can trigger in the TIME, there has been interest in combination therapies targeting both of these sequalae (77). Broadly speaking, logical categories of immunomodulators to preclinically study in combination with radiation for the treatment of Ewing sarcoma include: 1) immune checkpoint inhibitors (ICI), 2) cytokine modulators, and 3) cell-based therapies. Here we will briefly address each of these approaches.

The combination of radiation therapy and ICI in patients with advanced solid tumors has demonstrated promising early clinical results (74, 75). It has also been reported that the presence of DNA damage repair defects, such as germline BRCA 1/2 pathogenic variants, is correlated with increased expression of immunosuppressive ligands such as PD-1/PD-L1 (78), considered one marker of response to immune checkpoint inhibition. This association provides a rationale for preclinically testing the response of Ewing tumors with additional DNA damage repair defects to the combination of radiation and immune checkpoint inhibition.

In addition to examining ICIs, modulation of cytokines in the tumor microenvironment during radiation therapy is of great interest. While not every cytokine can be addressed in this mini-review, we will highlight two. TGF-β is an immunosuppressive cytokine that is increased in tumor microenvironments following radiation and has been shown to confer resistance to radiation (72). Inhibition of TGF-β during radiation has the potential to enhance the anti-tumor immune response (79). A second cytokine, IL-6, is known to be secreted by Ewing tumors (80, 81), and can be upregulated following radiation-induced DNA damage. Further, it is thought that the presence of IL-6 in the TME confers radiation resistance (82). IL-6 inhibitors are active in clinical trials as monotherapy for cancer (83), however, combination therapy with these inhibitors during radiation offers another therapeutic avenue worthy of preclinical testing.

Lastly, there is promise for delivering cell-based therapies in the setting of radiation. Chimeric antigen receptor T-cells (CAR-T) therapies have shown great success in the treatment of hematologic malignancies but have not seen the same success in solid tumors (84). Challenges have included identification of an ideal target antigen, cell trafficking to the tumor, and the overall immunosuppressive environment of solid tumors. Therapies targeting the DNA damage repair pathway have shown some success in solid tumors in improving response to CAR-T therapy through induction of a more pro-inflammatory TIME (85). Additionally, radiation therapy delivered prior to administration of CAR-T in a mouse model of glioblastoma demonstrated improved trafficking and efficacy of the CAR-T cells post-radiation (86). There is ongoing research in the field to identify a targetable antigen for cell based therapies for the treatment of Ewing sarcoma (87). In addition to CAR-T cell therapy, dendritic cell-based immunotherapy is a cell-based therapy that could logically be combined with DNA-damaging agents. Studies have demonstrated improved efficacy of dendritic cell vaccination when given in combination with radiation (88, 89). Lastly, as noted above, recent work has demonstrated the key role of NK cells in the radiation anti-tumor response. Understanding the role of NK cells in the TIME of Ewing tumors specifically during radiation is a priority (67, 90).

## Future directions and challenges

Significant historical impediments to studying the influence of DNA damage on the immune microenvironment in Ewing sarcoma include, but are not limited to, the lack of an immunocompetent animal model of Ewing sarcoma (4) and the sparsity of samples from disease relapse and pre-/post- intervention biopsies. Recently, a genetically engineered zebrafish model of Ewing sarcoma has been developed, which may offer a new immunocompetent model (91), although studies specifically investigating immune interactions in this model have not yet been performed. A potentially valuable model for studying the TIME of Ewing sarcoma is the development of humanized (presence of human immune cells), immunocompetent mouse models, a focus of ongoing work by our group. Developing and validating a robust preclinical model to study the impact of DNA-damaging agents used clinically for the treatment of Ewing sarcoma on the TIME is a crucial and necessary step toward determining promising immunomodulatory agents to partner with radiation or chemotherapy in an attempt to improve the outcomes for patients with advanced disease. While DNA damage, such as radiation therapy, is the focus of this mini-review, the impact of other novel agents, such as tyrosine kinase inhibitors, agents targeting EWSR1::FLI1, etc., on the Ewing sarcoma TIME are also worthy of exploration and represent a limitation of this mini-review.

## Author contributions

JD, AO, and KB contributed to the mini-review concept, initial manuscript writing, and editing. JD and KB generated the figures and completed formatting. All authors contributed to the article and approved the submitted version.

## Funding

KB is supported by the National Institutes of Health (K08CA252178) and Alex’s Lemonade Stand (Innovator Award). KB would like to thank the Morden Foundation, CHEMOWARRIOR, and the UPMC Children’s Hospital Foundation for their support. The University of Pittsburgh holds a Physician-Scientist Institutional Award from the Burroughs Welcome Fund (JD). Research reported here has been supported by the National Institutes of Health under their award number 5K12HD052892-15, PI: Terence S. Dermody, MD, JD and AO would like to thank Pittsburgh Cure Sarcoma for their support.

## Conflict of interest

AO receives research funding from Varian Medical Systems, Reflexion Medical and serves as a consultant for RenovoRx.

The remaining authors declare that the research was conducted in the absence of any commercial or financial relationships that could be construed as a potential conflict of interest.

## Publisher’s note

All claims expressed in this article are solely those of the authors and do not necessarily represent those of their affiliated organizations, or those of the publisher, the editors and the reviewers. Any product that may be evaluated in this article, or claim that may be made by its manufacturer, is not guaranteed or endorsed by the publisher.
